# Measuring the impact of spatial network layout on community social cohesion: a cross-sectional study

**DOI:** 10.1186/1476-072X-13-11

**Published:** 2014-04-11

**Authors:** Crispin H V Cooper, David L Fone, Alain J F Chiaradia

**Affiliations:** 1 Sustainable Places Research Institute, Cardiff University, 33 Park Place, Cardiff CF10 3BA, UK; 2 Institute of Primary Care & Public Health, School of Medicine, Cardiff University, Neuadd Meirionnydd, Heath Park, Cardiff CF14 4YS, UK; 3 School of Planning and Geography, Cardiff University, Glamorgan Building, King Edward VII Avenue, Cardiff CF10 3WA, UK

**Keywords:** Social cohesion, Severance, Walkability, Spatial network analysis, sDNA

## Abstract

**Background:**

There is now a substantial body of research suggesting that social cohesion, a collective characteristic measured by the levels of trust, reciprocity and formation of strong social bonds within communities, is an important factor in determining health. Of particular interest is the extent to which factors in the built environment facilitate, or impede, the development of social bonds. Severance is a characteristic of physical environments which is hypothesized to inhibit cohesion. In the current study we test a number of characteristics of spatial networks which could be hypothesized to relate either to severance, or directly to community cohesion. Particular focus is given to our most promising variable for further analysis (Convex Hull Maximum Radius 600 m).

**Methods:**

In the current study we analysed social cohesion as measured at Enumeration District level, aggregated from a survey of 10,892 individuals aged 18 to 74 years in the Caerphilly Health and Social Needs Cohort Study, 2001. In a data mining process we test 16 network variables on multiple scales. The variable showing the most promise is validated in a test on an independent data set. We then conduct a multivariate regression also including Townsend deprivation scores and urban/rural status as predictor variables for social cohesion.

**Results:**

We find convex hull maximum radius at a 600 m scale to have a small but highly significant correlation with social cohesion on both data sets. Deprivation has a stronger effect. Splitting the analysis by tertile of deprivation, we find that the effect of severance as measured by this variable is strongest in the most deprived areas. A range of spatial scales are tested, with the strongest effects being observed at scales that match typical walking distances.

**Conclusion:**

We conclude that physical connectivity as measured in this paper has a significant effect on social cohesion, and that our measure is unlikely to proxy either deprivation or the urban/rural status of communities. Possible mechanisms for the effect include intrinsic navigability of areas, and the existence of a focal route on which people can meet on foot. Further investigation may lead to much stronger predictive models of social cohesion.

## Background

There is now a substantial body of research that suggests that social cohesion, a collective characteristic measured by the levels of trust, reciprocity and the formation of strong social bonds within neighbourhoods or communities, is an important factor in determining health [1]. Of particular interest is the extent to which factors in the built environment facilitate, or impede, the development of social bonds, and so whether modifiable factors might be identified that are amenable to interventions to enhance social cohesion and health. This is an under-developed area of research and there is little evidence to inform policy.

Numerous aspects of the built environment could be hypothesized to affect social cohesion. One that has received frequent mention [2] is the degree of severance in the road and pedestrian network. Severance is loosely defined as the opposite of connectivity, although in the current context there is no universally accepted, formal definition of either of these terms. Intuitively we expect that networks with less severance will encourage more walking behaviour – creating more opportunities for people to interact with one another through chance encounters on the street – and also making it easier for people to visit one another either at home or in a public location. Thus, a community inhabiting a physically better connected network might exhibit stronger social connections between members of the community, while a network with a high level of severance may inhibit community cohesion.

The field of transport planning has concerned itself with the losses, as well as the gains in connectivity caused by new road developments since at least 1969 [3]. New road developments necessitate this kind of research as what to one person enables freedom, can to another mean confinement. A recent New Zealand report [2] reviewed the literature on community cohesion and severance, finding cohesion to be broadly defined as a form of social capital related to connectedness, and severance to be a either physical separation between people and facilities, or physical separation between people and other people. A UK report [4] defined severance differently, as a phenomenon encompassing not only physical and psychological barriers but also the social impacts of these. In this paper, we use the terms ‘severance’ and ‘connectivity’ to refer only to physical and psychological separation, and our focus is on measuring the social impact of these by studying community cohesion.

**Figure 1:**
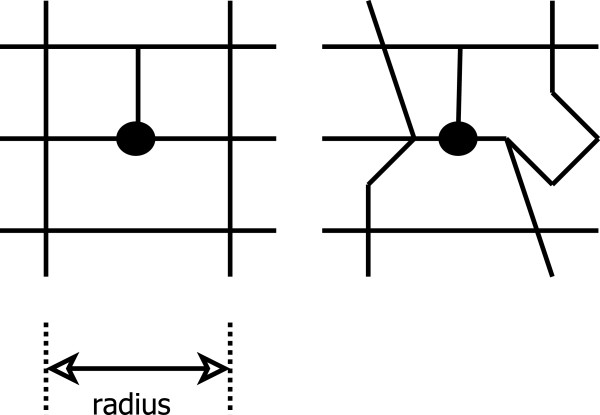
**Illustration of the limitations of intersection density as a measure for capturing network characteristics.** Both networks have 8 intersections within indicated network radius from central point.

A related body of work centres itself around the effect of neighbourhood walkability on social cohesion. Walkability is an aggregate measure which usually includes sub-variables such as street connectivity, residential density, land use mix and green spaces, availability of walking destinations and retail area [5-8]. It is notable that in these examples - and in many other studies of walkability without social cohesion – ‘street connectivity’ is measured only via intersection density, with higher densities being presumed to offer a greater choice of routes and hence walkability. Despite widespread use in the evaluation of urban designs, the effectiveness of this metric has recently come under criticism [9]. From our perspective, we note that it captures nothing of the shape of links between the intersections, nor the shape of the intersections themselves (Figure 1). Of the studies listed above, the only ones to find a quantifiable link between walkability and social cohesion were those that included a measurement of worthwhile walking destinations [6,7]; hence we suggest that intersection density alone is not a significant predictor of social cohesion.

Returning to the severance literature, although there is no unique definition, many suggestions are made for the measurement of physical severance which consider network characteristics in more detail [10]. One of these is mirrored directly in our own Spatial Network Design Analysis (sDNA) software [11], and is what we call the Diversion Ratio: “Compare the length of the direct route between the pairs of trip origins and their common destination ‘as the crow flies’, with the distance that the pedestrian will actually walk, taking account of development patterns”. Another suggestion given is to compare the physical area accessible from a given walking distance, to that which would be accessible if the pedestrian were to walk as the crow flies. For a given distance this is directly proportional to sDNA’s Convex Hull Area [12]. In total, sDNA computes 16 different localized network measures which could be hypothesized to affect community cohesion in a given area.

The aim of the present study is to determine whether any of the network characteristics we can measure are an important factor/associated with the degree of social cohesion in a community. We begin with an exploratory data mining exercise in which all 16 network metrics were tested for correlation with neighbourhood social cohesion using the Caerphilly Health and Social Needs Cohort Study, a community study of health inequality set in Caerphilly county borough, South Wales, UK [13]. We follow up with a validation test on a different dataset, in which community cohesion was measured for all Wales (though we exclude Caerphilly data from this analysis). Finally we present an extended analysis of the best performing metric on the Caerphilly dataset. The hypothesis is that built network effects - measured in some form - will correlate with social cohesion, both in isolation, and after controlling for social deprivation and the urban/rural status of communities. All of the parameters we test measure characteristics of the network which affect the frequency with which it is navigated on foot, and therefore the opportunity for interactions between people to take place, ultimately affecting community cohesion.

## Methods

### Study setting

The Caerphilly Health and Social Needs Cohort Study (CHSNS) is set in Caerphilly county borough, Wales, UK, a local government unitary authority of 28,000 hectares situated in the South Wales valleys with a 2011 census population of 178,800. Although the borough has some areas of outstanding natural beauty, located between the City of Cardiff to the south and the Brecon Beacons National Park to the north, there is a legacy of heavy industry. Local employment was historically dominated by the coal and steel industries, but a long period of decline led to major changes in the social and economic structure of the borough with high levels of unemployment and social deprivation. More recently, there has been substantial investment in regeneration activity in the most deprived areas of the borough.

In the present study we analysed data on social cohesion from the baseline survey we carried out in 2001. We sampled 17,979 individuals aged 18 to 74 years who were resident in the borough and obtained responses from 10 892 (60.6%) participants in a baseline postal questionnaire population survey [13]. Sampling was carried out stratified by the 36 electoral wards of local government in the study area (mean population 3600 adults) aiming to achieve an equal number of participants in each ward. Individual records were also linked to one of the 325 enumeration districts of residence defined in the 1991 UK Census using the address postcode. The enumeration district is a smaller geographically defined area than the electoral ward with an average population of 400 adults.

The dataset used for independent validation of the data mining results was drawn from data on 19,035 individuals combined from the other four available datasets in Wales that included questions on social cohesion: Living in Wales (2008), the National Survey of Wales (2009), the British Household Panel Survey (2008) and Understanding Society (2009).

### Social cohesion and deprivation data

**Figure 2:**
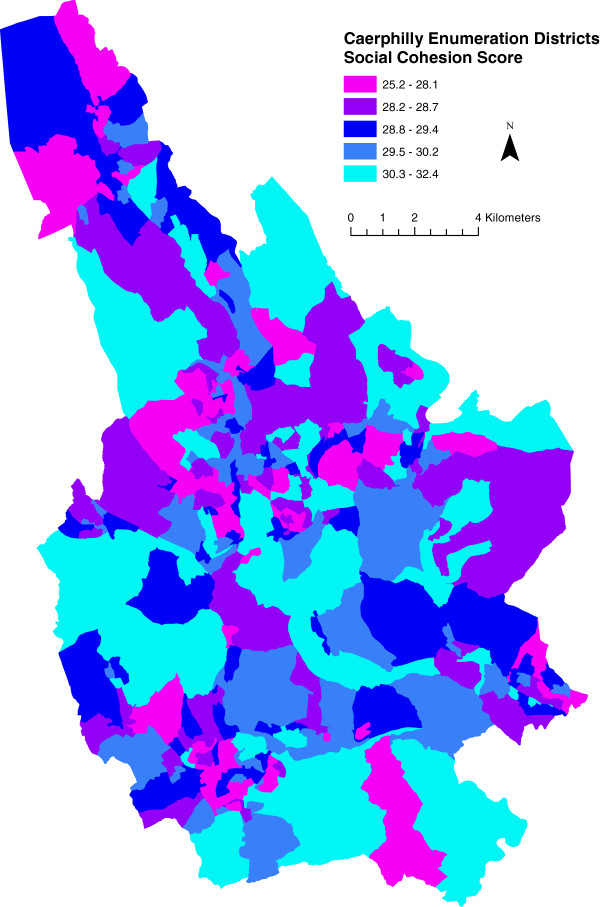
**Social cohesion scores for caerphilly county borough enumeration districts.** Legend class boundaries are set by quintile. Higher score implies more cohesion.

We have previously described the measurement of enumeration district social cohesion in detail [14]. In brief, for the CHSNS survey we included Buckner’s Neighbourhood Cohesion scale [15] in the questionnaire. We first carried out a factor analysis of individual responses that identified a social cohesion subscale measuring trust and reciprocity. We then carried out an ecometric analysis of individual responses to this subscale that suggested it could be used as a valid and reliable measure of social cohesion at enumeration district-level [14]. We then estimated mean enumeration district social cohesion scores. Figure 2 shows the geographical distribution of social cohesion in the borough.

**Figure 3:**
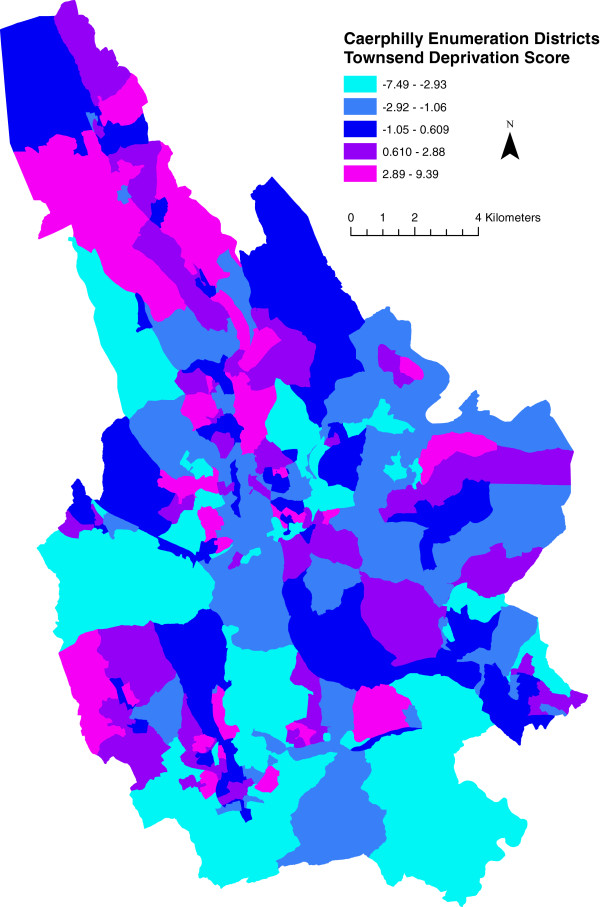
**Townsend deprivation scores for caerphilly county borough enumeration districts.** Legend class boundaries are set by quintile. Higher score implies greater deprivation. Note the colour scale is reversed as compared to all other figures to preserve the qualitative sense of the legend; that is to say, worse off areas appear in purple.

We measured enumeration district social and material deprivation using the well-established Townsend Index [16] with scores for Caerphilly borough shown in Figure 3. Urban/rural classification of areas was taken from the definitions and data of the 1991 census [17] and coded as a binary variable.

In the all-Wales dataset, we measured social cohesion at LSOA-level using the same methods, estimating the mean 2001 Census lower layer super output area (LSOA) score from responses to the Buckner’s Neighbourhood Cohesion scale.

### Spatial network analysis

Network analysis was conducted using our own general-purpose software sDNA which we make freely available online [11].

All networks are made up of a set of nodes connected together by links - in the case of a road network, nodes correspond to junctions or intersections between roads. In *spatial* networks, nodes always have physical positions, and links always have a physical shape - in contrast to e.g. a social network in which links can represent abstract concepts such as acquaintance. The key idea of spatial network analysis is to create statistics that describe the configuration of any given network.

A richer analysis is possible, however, if we create statistics for each *part* of the network individually, that describe its relationship to the surrounding network. Such measures lend themselves well to spatial analysis studies, when combined with other data which varies across space. In the current case, we examine how these measures applied to the road network correlate with social cohesion.

A key component of our methodology is to standardize on the network link as a unit of analysis unless there are good reasons for doing otherwise. In other words, the *parts* into which we divide the network are individual links. A link is defined as the connection between two adjacent junctions, or between a junction and a dead end, such that there are no junctions in between: until the end of the link is reached, the movement choices of the user are by definition restricted to (i) continuing forwards, (ii) turning around or (iii) leaving the spatial network altogether. Links are in some sense, an atomic component from which networks are built, so standardizing on them sidesteps the problem of modifiable spatial units of analysis, at least at the network level – a method not possible, at least on geographic scales, with areal units [18,19].

Specifying that the network link is our primary unit of analysis may seem redundant without mentioning the existence of alternatives – dividing the network into equal units of length, for example, or analysing population-weighted address points attached to the network rather than the network itself. The latter is indeed a reasonable, though data heavy approach (and would miss built environment features other than residences). In the absence of such data however, network link density tends to proxy interesting activity of some sort, hence the drive towards standardization on this unit. Specifically in the case of a road network, link density can correlate as much as 99% with the density of jobs and homes [20].

Another key component of our analysis is to choose a scale of interest. This defines how much of the ‘surrounding network’ we consider when computing descriptive statistics for each individual link. As we are studying community cohesion, this scale is likely to match sensible walking distances: up to 1.5 km, but usually less. The distance used is referred to as the *network radius*, in the sense that all the links analysed surrounding any given origin, fall within^a^ the given radius of that origin, with distance measured not as the crow flies but via the shortest route possible along the network.

Once the ‘surrounding network’ has been defined for each link, we must analyse it. Many of the statistics we produce are computed by considering shortest paths between pairs of links in the surroundings. For this part of the analysis we have chosen to focus on angular shortest paths (in contrast to the Euclidean shortest paths used to define the surroundings). This means that routes are chosen based on minimizing the angular change – the cumulative angle turned on corners and at junctions - rather than minimizing the Euclidean distance travelled. Angular analysis is believed to reflect the cognitive difficulty inherent in navigating [21,22], so could be thought to be more relevant for capturing subtleties in the layout and the navigability of areas. It reflects an assumption that people prefer simple, rather than complex routes.

The 16 statistics we compute are described in more detail in Table 1.^b^ As all statistics are produced per individual link, these must be aggregated to the areal units of the cohesion data by taking a mean value for each areal unit. Following the discussion above, this is averaged over links and is not weighted by the length of links. Areal units are also buffered by 30 m so as not to exclude the effect of links which form the boundary between two districts (as is often the case); such links will therefore be counted reliably in all neighbouring districts rather than unreliably in only some of them.

**Table 1:** Variables tested during data mining process

**Name (sDNA abbrev.)**	**Description**	**Hypothesis (see Table **2**)**	**Best correlation (Pearson’s r) and radius**
Number of links in radius (Links)	~	D	-0.051 1500 m
Network length in radius (Length)	~	D	+0.029 300 m
Network quantity penalized by angular distance (NQPDA)	Number of links in radius penalized by angular distance to each	D,DD,T	-0.039 1500 m
Two phase destination angular (TPDA)	Measure of destination ‘popularity’ under TPBtA model	D	+0.083 1500 m
Mean geodesic length angular (MGLA)	Mean network Euclidean length (in metres along network) of routes to all destinations in radius	DD,T	-0.149 600 m
Mean crow flight distance (MCF)	Mean Euclidean length (in metres as the crow flies) of routes to all destinations in radius	DD	+0.143 1200 m
Mean angular distance (MAD)	Mean angular length (in degrees) of routes to all destinations in radius	DD,T	-0.079 600 m
Mean diversion ratio angular (DivA)	Mean of network length/crow flight length per route	T	-0.116 1500 m
Angular betweenness (BtA)	Flow prediction based on angular shortest paths not exceeding radius	F	+0.026 300 m
Two phase angular betweenness (TPBtA)	As BtA but limiting trip generation to a fixed amount per origin distributed over all destinations in radius	F	+0.025 1500 m
Junctions in radius (Jnc)	~	L	-0.047 1500 m
Connectivity in radius (Con)^a^	Total number of link ends joining junctions in radius	L	-0.050 1500 m
Convex hull area (HullA)	Area of convex hull formed by all points in radius	E	+0.129 1200 m
Convex hull perimeter (HullP)	Perimeter of convex hull	E	+0.191 1200 m
Convex hull max radius (HullR)	Maximum radius of convex hull *(seeDiscussionfor more information)*	E	+0.251 600 m
Convex hull shape index (HullSI)	‘Circularity’ of convex hull shape	E,H	+0.089 300 m

^a^Connectivity in radius’ here refers to a specific use of the word ‘connectivity’ relating to counting link ends. In the remainder of the paper, ‘connectivity’ retains its broader definition. Full descriptions of all measures in this table are available in [12].

### Data mining

During the data mining phase of the study, a variety of network variables computed by sDNA were tested for their correlation to community cohesion. Tables 1 and 2 list the variables tested and the reasons a correlation may be expected in each case. We test each variable over five different scales of interest (network radii): 300 m, 600 m, 900 m, 1200 m and 1500 m. These provide a broad spread over sensible walking distances.

**Table 2:** **Hypothesis referenced in Table**1

D (Density)	These are all measures of built environment density. Hypothesis: there is an optimum built environment density for social cohesion, sufficient to ensure interactions between residents but possibly with diminishing returns in high density areas as the number of familiar individuals is diluted in the unfamiliar population.
DD (Density distribution)	These are all measures of how the built environment is distributed within the radius: close to or far from the origin. Hypothesis: there is an optimum distance to neighbouring dense areas for social cohesion, striking a balance between accessibility to community focal points and isolation from problems associated with busy areas.
T (Twistiness)	These are all measures of how ‘twisted’ the local network is. Angular distance proxies cognitive difficulty of navigating a route. Hypothesis: ‘twistier’ environments impose a greater psychological barrier between each origin and its neighbourhood. Again there should be an optimum barrier that strikes a balance between access to positive effects and isolation from negative.
F (Flow)	These are both estimates for pedestrian flow under different models. Hypothesis: there is an optimum level of pedestrian flow for community cohesion. More flow creates more opportunities for community-strengthening interactions, while too much dilutes community effects.
L (Literature)	These measures reflect the dominant method in the literature of measuring connectivity via intersection density. Hypothesis: more junctions in an area make it more navigable for pedestrians; therefore it is more frequently navigated on foot, creating opportunities for pedestrian interaction.
E (Efficiency)	These measures reflect the efficiency of the network for covering either space or distance in the local area. Thus they are a more sophisticated measure of navigability, which takes into account the shape and arrangement of links as well as the raw number of connections. Hypothesis: greater efficiency of navigation on foot will lead to more frequent navigation on foot, creating more opportunities for pedestrian interaction.
H (Homogeneity)	This measure represents the degree to which the local network ‘looks the same’ in all directions. Hypothesis: that variety in the local area can foster a greater sense of social cohesion due to the sense of identity associated with living somewhere unique.

The aim of the data mining was to identify the most promising variable for further analysis. To validate the choice of such a variable, a test of the same variable was performed using an independent data set.

### Further investigation

The best performing variable – HullR600c – was investigated further. To mitigate the concern that HullR is a proxy for either deprivation (via the social characteristics of residents in different designs of housing estate), or an urban/rural divide (as rural areas will tend to have higher HullR due to the small size of settlements and hence the proximity of all residents to a long and relatively straight connecting road), we conducted three bivariate ordinary least squares regressions (of cohesion vs deprivation, HullR600c and urban/rural status in turn). We then conducted a multiple ordinary least squares regression of cohesion against all other parameters together.

As social cohesion is of greater health importance to the most deprived communities [23], we chose to investigate how the effect of spatial layout varies on communities with differing deprivation levels, by re-running the multivariate regression model for each tertile of deprivation individually. Finally, bivariate correlations of HullR600c were tested against social cohesion for varying sizes of network radius, thus informing the choice of a suitable scale for future studies.

Statistics were computed using the open-source Python *statsmodels* and *pandas* packages.

## Results

### Data mining and validation

In total, 16 variables were tested over 5 different spatial scales. Viewed conservatively, this constitutes a test of 16 × 5 = 80 variables; thus, applying Bonferroni correction to our results suggests that p-values should be multiplied by 80. It is noted that less strict approaches are possible: in particular, the testing over multiple scales can be considered as a calibration exercise for each parameter in which case the effective number of tests is reduced to 16. Also, correlations between different scales of the same parameter, as well as occasionally between different parameters, mean that some of the multiple tests are not independent. As our results remain statistically significant under the most conservative interpretation, we do not explore this further. The variable which correlated most highly with social cohesion was Convex Hull Maximum Radius for a network radius of 600m (HullR600c^c^), with bivariate correlation coefficient r = 0.251 and p-value <0.001 (precisely p = 4.65 × 10^-6^).

Table 3 summarizes these results alongside those from the validation test on the all-Wales (excluding Caerphilly) dataset. HullR600c remains valid (r = 0.11, p = 9.46 × 10^-6^). The weaker effect size is to be expected as (i) sampling of social cohesion in the all-Wales survey is more sparse and hence more prone to sampling variability; (ii) cohesion is measured at LSOA rather than ED level, thus hiding differences that occur on a fine spatial scale and diluting the analysis.

**Table 3:** Results of mining and validation correlation tests for HullR600c

**Test purpose**	**Mining**	**Validation**
Dataset	Caerphilly	All Wales excl. Caerphilly
Spatial unit	ED	LSOA
No. observations	325	1742
Pearson’s r (HullR600c vs social cohesion)	0.251	0.106
p value	4.65 × 10^-6^	9.46 × 10^-6^

Table 4 shows the overall r^2^ for the four regression models (three bivariate and one multiple). On its own, HullR600c correlates with cohesion with r = 0.25. Details from the multiple regression model are shown in Table 5. The effect of HullR remains similar, with a standardized coefficient of 0.26 and a high level of significance; this is outweighed only by deprivation which has a stronger negative effect on social cohesion.

**Table 4:** Regression model summary

**Model**	**r**^**2**^
Cohesion ~ deprivation + HullR600c + urban	0.249
Cohesion ~ deprivation	0.167
Cohesion ~ HullR600c	0.063
Cohesion ~ urban	0.013

**Table 5:** Cohesion vs deprivation, HullR600c and urban/rural regression model

No. observations	325		
Independent Variables	3		
r^2^	0.249		
Adjusted r^2^	0.242		
Variable	Deprivation	HullR600c	Urban
Standardized coefficient	-0.42	0.26	-0.10
Standard error	0.048	0.048	0.048
t statistic	-8.69	5.44	-2.04
p value	1.9 × 10^-15^	1.1 × 10^-7^	0.042

**Figure 4:**
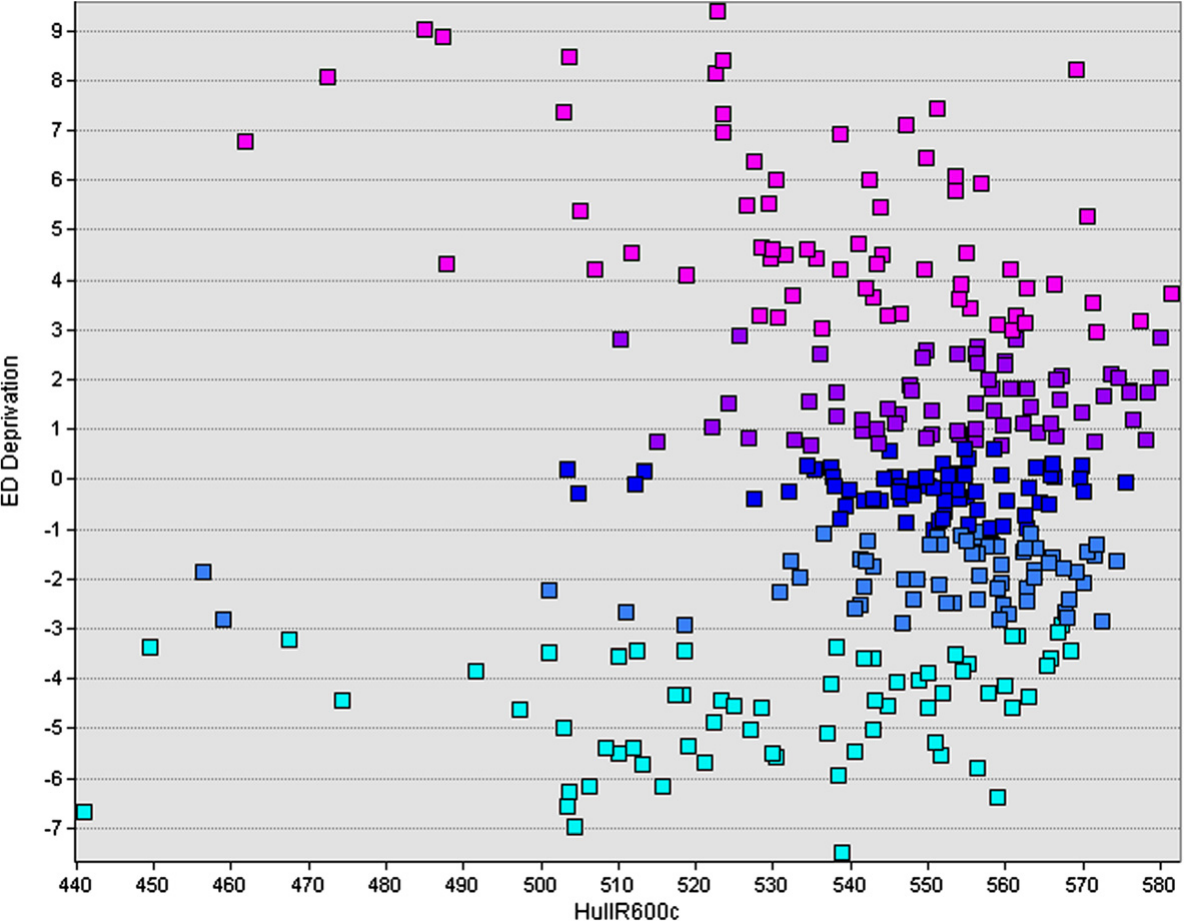
**Scatterplot of deprivation vs convex hull maximum radius.** The colouring is according to deprivation quintile, illustrating that areas which are outliers low in physical connectivity are either very poor or very rich.

The test of social cohesion against each tertile of deprivation individually is shown in Table 6. The most significant association between HullR and cohesion occurs for the most deprived tertile (standardized coeff. = 0.41). Deprivation is still included as a regressor in the model for each deprivation tertile, as an interesting U-shaped relationship seems to exist connecting HullR to deprivation (Figure 4). Deprivation within each tertile is not significant at the 5% level, thus we conclude that HullR is unlikely to be a proxy for deprivation.

**Table 6:** Cohesion vs HullR600c and urban/rural regressed for each tertile of deprivation

			**Standardized coefficient (p value in brackets)**		
**Deprivation tertile**	**r**^**2**^	**r**	**HullR600c**	**Deprivation**	**Urban**
0 (least deprived)	0.08	0.28	0.21 (0.009)**	-0.32 (0.154)	-0.08 (0.375)
1	0.18	0.42	0.31 (0.016)*	-0.65 (0.056)	-0.24 (0.005)**
2 (most deprived)	0.20	0.45	0.41 (0.000)**	-0.13 (0.472)	-0.02 (0.846)

*Significant at 5% **significant at 1%.

**Figure 5:**
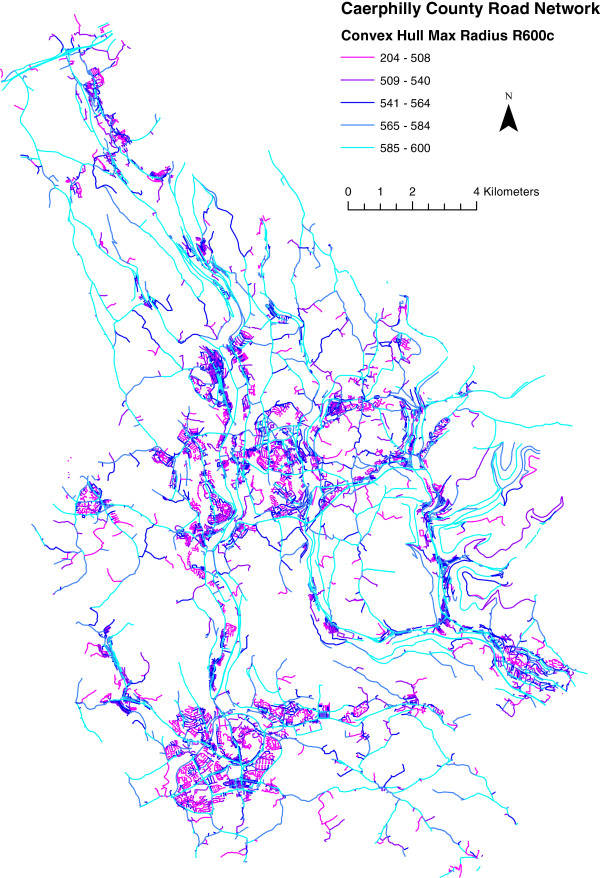
**Maximum convex hull radius in metres for links of caerphilly county borough itn road network.** this represents the maximum distance in metres, as the crow flies, obtainable from the angular centre of each link by traversing 600 m along the road network. computation was performed with a 3 km buffer around the area (not shown) to remove edge effects. legend class boundaries are set by quintile.

**Figure 6:**
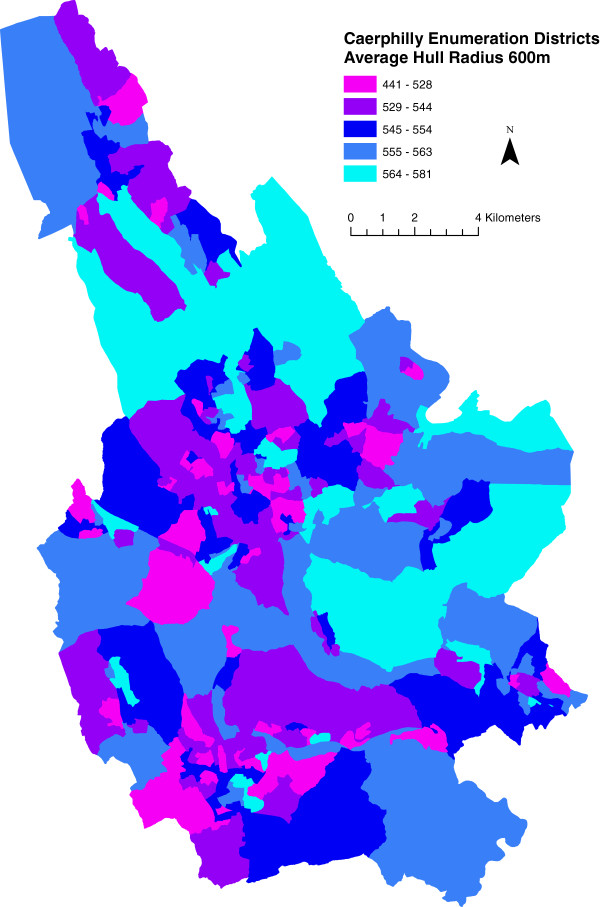
**Average convex hull maximum radius in metres of caerphilly county borough enumeration districts.** This represents the mean value, for all network links in each area, of the data displayed in Figure 5. Legend class boundaries are set by quintile.

**Figure 7:**
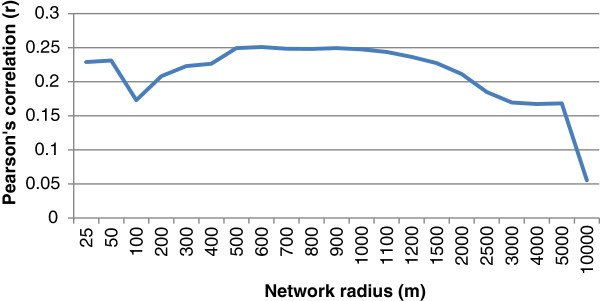
Bivariate correlation of hullr with social cohesion for varying radii.

Figures 5 and 6 show maps of HullR600c for the Caerphilly county borough road network, and Enumeration Districts respectively. Finally, Figure 7 shows how the correlation between HullR and social cohesion varies with scale.

## Discussion

**Figure 8:**
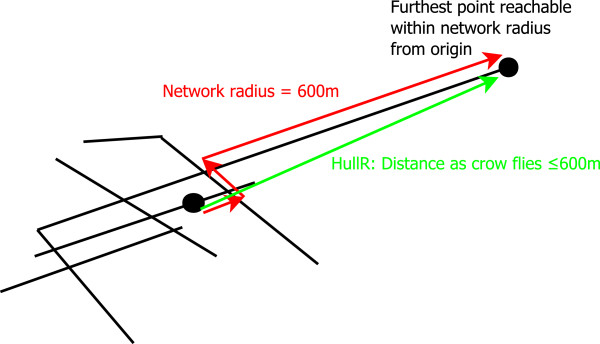
**Illustration of HullR600 parameter.** We measure the maximum crow flight distance achievable by traversing a fixed network distance.

The most promising parameter discovered during the data mining phase was HullR600c, most simply defined as ‘the greatest distance, as the crow flies, from the centre of each link, that we can obtain by traversing 600 m along the network’ (Figure 8). Thus, higher values of HullR imply that the nearby network covers Euclidean distance more efficiently – measuring something akin to the inverse of physical severance. The terminology arises because the measured distance is the same as the maximum radius of a convex hull of all points falling inside the network radius (if we define the hull’s centre as the centre of the origin link). This is admittedly a trivial point, but useful because it relates the measure we use to other spatial network statistics also based on convex hulls.

Figure 5 shows the HullR600c parameter as computed for all links in the Caerphilly county borough road network. In general, the figure shows how different urban forms are characterised differently by HullR, although within the confines of this model, the lowest ‘severance’ is in fact exhibited by major roads. This observation is at odds with the literature, which emphasizes the divisive effect that major roads can have on communities due to the physical and psychological barrier associated with heavy traffic flow [2-4]. Our intention is not to contradict the literature on this point, so much as to illustrate a potential limitation of HullR600c as a measure of severance for individual road links rather than the larger areal units that we use in this paper. In an analysis based entirely on HullR, major roads do – counterintuitively – appear to reduce severance; in reality this is not likely to be the case, as could likely be illustrated by a model which explicitly includes traffic flow. Such a model is not necessary in the current study because the effect of these major roads is greatly diminished when HullR600c is aggregated to the areal units of analysis.

Another notable feature of Figure 5 is that some quiet housing estates composed mostly of cul-de-sacs exhibit a high degree of severance. This reflects (i) as already stated, the fact that we do not explicitly model traffic flow; and (ii) that our spatial model does not include pedestrian footpaths. This is likely to exaggerate the severance in housing estates which sometimes include pedestrian links between different vehicular cul-de-sacs. However, it may also be indicative of success in capturing pedestrian severance in housing estates designed with the private car in mind.

Once HullR is averaged over enumeration districts (Figure 6), the picture of severance changes. The low HullR scores for major roads become largely irrelevant due to our policy of averaging over network links rather than network length. This favours inhabited areas over uninhabited ones, and hence is intuitively a better representation of severance in the residential environment than Figure 5.

Figure 7 shows a plot of the association between cohesion and HullR for varying values of the network radius. As expected, the network radii which show the strongest link with community cohesion are those which are an appropriate length for pedestrian trips, with the maximum correlation occurring at 600 m. This is a realistic size for walkable catchments, with 600 m corresponding to a 5–10 minute walk for most people [24]. Overall this gives confidence that the causal mechanism for the influence of HullR on cohesion does indeed relate to pedestrian accessibility. There is a secondary peak in association for very short radii (<50 m) which is likely to indicate more about the shape of individual roads than the connectivity of neighbourhoods. Thus it is possible that on very short scales this variable may be a proxy for specific types of spatial design, which in turn are associated with specific demographics, and hence the presence (or absence) of deprivation. In a social cohesion context this secondary peak is not of interest to us.

### Strengths and limitations

This study has investigated a number of network characteristics that could be hypothesized to have an effect on community social cohesion, and has shown that maximum convex hull radius 600 m has a small but statistically significant effect. In other words, higher social cohesion tends to arise in built environments which allow more efficient traversal on foot on a 600 m scale. This holds both in an independent test, and a multiple regression analysis including deprivation and a measure of urban/rural status. The measure of social cohesion used is well-validated and the measure of social deprivation is well-established, and the study is conducted on a fine spatial scale.

That this link has been established is an improvement on prior studies of the effect of spatial network design on community cohesion, which either find no link [5,8] or do find a link but this is likely related to considering walking destinations [6,7], which we do not take into account here. We suggest that this is because our network measure considers network geometry in greater detail than junction density alone.

Splitting the analysis by tertile of deprivation, HullR600c was the only variable in a network, cohesion, deprivation and rurality model to have a consistent effect across all three deprivation levels, with a particularly strong effect on the most deprived communities. Interestingly, this mirrors the finding that the health benefits of social cohesion itself are strongest in the most deprived communities [23]. Thus, further investigation of underlying mechanisms may lead towards urban designs which assist cohesion and its associated health benefits, and the reduction of health inequalities.

One limitation of the study is that it does not include levels of road traffic, which are thought to contribute to severance. This in itself would be worthwhile further work; however, as long straight roads typically carry more traffic, inclusion of traffic variables is likely only to strengthen the association between cohesion and convex hull maximum radius. A third limitation is the lack of a detailed causative mechanism for the link between HullR and cohesion. Thus, it is expected that detailed investigation of this mechanism will lead to the discovery of measures which have a stronger influence on social cohesion than HullR does.

A final point of caution is that HullR is not a measure of the quantity of destinations within a given distance; nor is it intended to be. It tells only about the accessibility of those destinations which are available, no matter how many or few there may be. Intuitively, it would make a lot of sense that worthwhile walking destinations are an essential ingredient of walkability – a point that appears to be confirmed by [5-8]. Therefore we suggest that while HullR is an important factor affecting cohesion, it is not a metric that should be used in isolation.

### Possible mechanisms

The biggest question for further research, then, is exactly what causes this curious link between geometry and community. A central tenet of spatial network analysis is the idea that a vast wealth of information lies encapsulated in the layout of spatial networks; in the current case a small correlation could perhaps be the metaphorical tip of an iceberg – that is to say, a much better predictive measure could be found, if only we could unearth more detail on the precise causal mechanism. Much information is lost when a detailed network layout is condensed to a single regression variable, so future analysis will require different techniques.

Two ideas then, for plausible general mechanisms are as follows. First, there is the hypothesis that led us to test HullR in the first place: that it may directly represent the intrinsic navigability, on foot, of an area. An area more navigable on foot is, all other things being equal, more likely to be navigated on foot, leading to myriad opportunities for chance encounters between residents; the forming of friendships and ultimately the strengthening of community cohesion. A second interesting possibility, however, is that high values of HullR indicate the existence of a long, straight road somewhere in a given neighbourhood: - pedestrian routes which first access this road then proceed along it will rapidly progress away from their origin, leading to high values of the HullR statistic. Such a road may or may not be the high street of a town; it may indicate a “main street” of a small district, or even just a convenient route through an area which in some sense becomes a “focal route” for a community. This route would itself provide more opportunities for interaction between people, ultimately leading to increased cohesion. Both of these hypotheses are compatible with literature in which spatial cohesiveness in communication communities has been observed at the shortest spatial resolutions available from mobile phone network data analysis [25,26].

One salient feature of HullR is that, despite our choice to focus on angular analysis, the measure is independent of whether shortest paths are defined by Euclidean or angular metrics^d^. This may indicate that angular analysis is not so relevant for users who are highly familiar with an area, and hence ‘know all the short cuts’.

Another important point for discussion is that HullR is a measure not of the average route through an area, but of the most efficient possible path from a given origin, with the destination chosen freely to maximise the efficiency of the path. This is something that sets it apart from the other measures of network efficiency we tested - which tend to measure either average routes to all destinations (e.g. diversion ratio) or the average of the best routes in each possible direction (e.g. convex hull area) - as well as a departure from literature recommending that connectivity measures should be omnidirectional [9]. We suggest two possible hypotheses for the success of HullR in spite of this seeming limitation.

One explanation is that while *multi*directionality is important, *omni*directionality may not be (residences on the edge of a settlement may after all exhibit good social cohesion despite lacking connectivity beyond the settlement edge). Thus omnidirectional measures such as HullA do not outperform the unidirectional HullR, especially if collinearity between these two measures helps to compensate for HullR’s unidirectionality (on the Caerphilly road network HullA and HullR are correlated with r = 0.47; it would be interesting to see how this varies in other areas). Nonetheless, a measure which strikes a balance between uni- and omnidirectionality may outperform either in isolation.

The other explanation is that multidirectionality may not be important at all: although good accessibility to a community focal point or focal route is important, the average residence may need accessibility only in the direction of that focal point/route, with multidirectionality being important only for the focal point/route itself. Here we should note that community facilities as destinations are not explicitly included in our model; thus, perhaps the success of HullR can be explained by assuming that such focal points and routes are already, either by design or through economic natural selection, situated on the best routes ‘discovered’ by HullR. If this is the case, then it would represent a strength of HullR as a tool to guide urban designers when they have no control over facility placement, although it would be an additional design constraint when they do: in the former circumstance we can only hope that facilities will end up in optimal locations, while in the latter we cannot assume this as it is our own job to ensure it! Relating to this point, it should be noted that HullR as a measure for evaluating designs could easily be susceptible to ‘gaming’ by developers who have different objectives to the regulatory body. Future work aiming to develop robust evaluation metrics would need to take this into account.

It is of course possible that the reported link between HullR and cohesion is simply a proxy for something else specific within the confines of the study area. This would not be a useful finding as we are more interested in the general relationship. Within the constraints of the current study we have attempted to answer this concern (i) through multivariate models including deprivation and urban/rural classification, (ii) through validation on an independent data set and (iii) through a multi-scale analysis that demonstrates the highest association occurring at pedestrian scales of interest. These tests, along with the severance literature, hint that the mechanism is likely to be related to general pedestrian activity.

## Endnotes

^a^Note that while the unit of analysis is the complete network link, where a link crosses outside of a network radius from any origin, sDNA can be configured to include the portion of the link that falls inside the radius (albeit with a smaller weighting to reflect the fact that it is a partial link). We refer to this as our *continuous space* algorithm. Continuous space is useful for preserving accuracy on short (i.e. pedestrian) scales.

^b^A more detailed description of the most promising statistic discovered during data mining (HullR600c) is also given in the Discussion section.

^c^The ‘c’ suffixing HullR600c indicates continuous space analysis.

^d^ …almost! Strictly speaking, sDNA will by default perform analysis from the centre of each link, using either the angular or Euclidean centre depending on the type of analysis. Thus none of the measures described here are completely independent of analysis type, but for HullR in the current study the differences are negligible.

## Abbreviations

CHSNS: Caerphilly Health and Social Needs Cohort Study; ED: Enumeration district; sDNA: Spatial design network analysis; LSOA: Lower super output area; HullR: Convex hull maximum radius; HullR600c: Convex hull maximum radius for 600 m continuous network radius; OS ITN: Ordnance Survey integrated transport network.

## Competing interests

Crispin Cooper is lead developer and Alain Chiaradia is concept lead for the sDNA software, which we make available to all users free of charge. If a commercial extension to the software were to be produced in future, they would have a financial interest in it.

## Authors’ contributions

CC developed the network analysis software, derived descriptive network variables and performed the analysis. DF prepared the health dataset for analysis. AC provided the original concept for the sDNA software (including in particular the concepts of link-based and convex hull analysis) and assisted with contextualisation of results. CC wrote the first draft of the paper and all authors edited and approved the final version.

## Authors’ information

Crispin Cooper is a research associate at Cardiff University Sustainable Places Research Institute, where he develops the sDNA network analysis software and researches applications of spatial and network analysis in sustainability science. He was awarded his PhD in City & Regional Planning by Cardiff University in 2010 and his undergraduate degree in Computer Science from Cambridge in 2002. In the interim he worked for the Intelligent Systems group at the University of York.

David Fone is professor of health sciences research in the Institute of Primary Care & Public Health in the School of Medicine, Cardiff University and a member of the Sustainable Places Research Institute. He is principal investigator of the Caerphilly Health & Social Needs Cohort Study.

Alain Chiaradia is a lecturer in urban design at the School of Planning and Geography, Cardiff University. He has 15 years’ experience in operational research using spatial design network analysis.
